# Albumin Nanoparticle Formulation for Heart-Targeted Drug Delivery: In Vivo Assessment of Congestive Heart Failure

**DOI:** 10.3390/ph14070697

**Published:** 2021-07-19

**Authors:** Nikita Lomis, Ziyab K. Sarfaraz, Aiman Alruwaih, Susan Westfall, Dominique Shum-Tim, Satya Prakash

**Affiliations:** 1 Department of Biomedical Engineering, Biomedical Technology and Cell Therapy Research Laboratory, McGill University, 3775 University Street, Montreal, QC H3A 2B4, Canada; nikita.lomis@mail.mcgill.ca; 2 Division of Experimental Medicine, McGill University, 1001 Boulevard Décarie, Montréal, QC H4A 3J1, Canada; 3 Division of Cardiac Surgery and Surgical Research, McGill University, 1001 Boulevard Décarie, Montréal, QC H4A 3J1, Canada; Ziyab.Sarfaraz@mail.mcgill.ca (Z.K.S.); Aiman.Alruwaih@mail.mcgill.ca (A.A.); Dominique.Shum-Tim@mcgill.ca (D.S.-T.); 4 Meakins Christie Laboratories, Department of Microbiology and Immunology, McGill University, 1001 Boulevard Décarie, Montréal, QC H4A 3J1, Canada; Susan.Westfall@mcgill.ca

**Keywords:** albumin, nanoparticle, protein, milrinone, heart failure, drug delivery, pharmacokinetics, biodistribution

## Abstract

Congestive heart failure is a fatal cardiovascular disease resulting in tissue necrosis and loss of cardiac contractile function. Inotropic drugs such as milrinone are commonly used to improve the myocardial contractility and heart function. However, milrinone is associated with severe side effects and lower circulation time. In this article, a novel protein nanoparticle formulation for heart-targeted delivery of milrinone has been designed and tested. The formulation was prepared using albumin protein conjugated with the targeting ligand, angiotensin II peptide to form nanoparticles following the ethanol desolvation method. The formulation was characterized for size, charge, and morphology and tested in a rat model of congestive heart failure to study pharmacokinetics, biodistribution, and efficacy. The overall cardiac output parameters were evaluated comparing the formulation with the control non-targeted drug, milrinone lactate. This formulation exhibited improved pharmacokinetics with a mean retention time of 123.7 min, half-life of 101.3 min, and clearance rate of 0.24 L/(kg*h). The targeted formulation also significantly improved ejection fraction and fractional shortening parameters thus improving cardiac function. This study demonstrates a new approach in delivering inotropic drugs such as milrinone for superior treatment of congestive heart failure.

## 1. Introduction

Cardiovascular diseases are responsible for disability and mortality across the developed and developing world, of which congestive heart failure (CHF) has ever-rising incidences [1]. The more commons treatments for CHF include surgical measures such as heart transplant, ventricular assist devices, bypass surgeries, and stents [2]. Medical treatments involve delivery of drugs such as inotropes, angiotensin-converting enzyme inhibitors, beta blockers, diuretics, etc. To improve the limitations associated with the use of non-targeted drugs, promising research has been undertaken especially in the field of nanomedicine [3,4]. The application of nanomedicine in cardiac therapy represents an innovative approach for efficient delivery of therapeutic agents such as drugs, genes, growth factors, cytokines, and other molecules [5,6,7,8]. However, despite the prominent advances on the use of nanoparticles as drug delivery systems for cancer therapy, fewer such studies have been reported in cardiovascular diseases, which claim millions of lives annually, also causing a huge economic burden [9,10]. Therefore, developing a targeted nanoparticle formulation would help overcome limitations associated with current pharmacological treatments and result in effective treatment of CHF [11].

Currently, the treatment of myocardial infarction (MI) and CHF involves administration of drugs either intravenously or orally in both adults and pediatric patients. However, lower retention times call for a continuous infusion of drugs with lack of target specificity, potentially causing toxicity and other side effects such as arrythmias, palpitations for off-target organs [12]. Though some studies using nanoparticles for growth factor delivery have suggested strong cardioprotective effects through direct myocardial injection, retaining the nanoparticles remains a challenge [6,13,14]. An ideal nanoparticle formulation would be biocompatible, biodegradable, possessing optimal particle size, surface charge, surface modifiable characteristics, targeting features, and longer blood circulation time. The nanoparticle system reported in this study, developed from human serum albumin (HSA-NPs) protein covers the above aspects and also binds the milrinone (MRN) drug molecule [10]. With an improved pharmacokinetics profile and drug delivery to the infarcted site, it enhances the therapeutic effect of the drug. Further, the controlled release of the MRN from the nanoparticles at the targeted site would allow for a single dose injection as opposed to a continuous supply. Being biodegradable, non-toxic, and non-immunogenic, HSA-NPs are a more favourable option than other carriers [15]. Several albumin-based nanocarriers have already been developed owing to their increased specificity, biocompatibility, and ability for surface functionalization [5,16].

It is widely known that under MI and CHF, the angiotensin II type 1 (AT1) receptors are overexpressed on the myocardium. These receptors are specific for the angiotensin molecule, an 8-amino acid chain peptide (Asp-Arg-Val-Tyr-Ile-His-Pro-Phe) [17,18]. The overexpression of AT1 receptors has been correlated directly with cardiac remodelling due to HF, explaining the use of drugs to block these receptors [19]. Similarly, the downregulation of AT1R expression has shown significant improvement in cardiac function [7,20]. Emerging strategies on functionalized AT1 receptor-targeted nanoparticles such as liposomes, quantum dots, PLGA microcapsules as drug delivery systems present a creative and modern outlook on heart failure treatment [8,16,19]. Thus, the study of AT1 receptors as targets for the delivery and uptake of drugs, nanoparticles, and other biomolecules has immense potential for development for potent cardiac therapies.

In this study, we report the design and development of a targeted nanoparticle formulation for delivery of MRN to treat CHF. This formulation was prepared using HSA conjugated with the targeting ligand, AT1 peptide to form nanoparticles binding MRN. The AT1-HSA-MRN-NPs were characterized in vitro and tested in vivo. We studied the pharmacokinetic parameters of the AT1-HSA-MRN-NPs compared with the control non-targeted drug, MRN Lactate. Through a second in vivo study, the treatment efficacy of the AT1-HSA-MRN-NPs vs. MRN Lactate has been evaluated in a rat model of congestive heart failure.

## 2. Results

### 2.1. Synthesis of AT1-HSA-MRN-NPs

**Figure 1:**
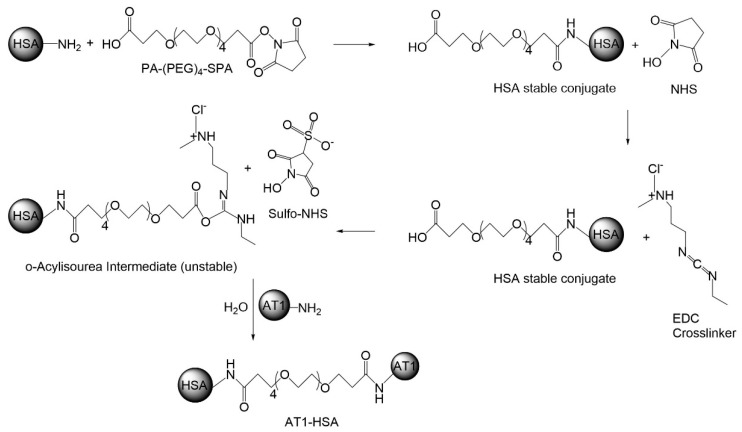
Schematic representation of the surface modification of the HSA molecule for binding with the AT1 peptide through a two-step chemical conjugation reaction using cross-linkers PA-(PEG)_4_-SPA and EDC/Sulfo-NHS.

The AT1-HSA-MRN-NPs were developed for targeted delivery of MRN to the heart [10]. The overexpression of the AT1 receptors in cardiomyocytes allows for improved targeting of the AT1 peptide and hence greater uptake of the nanoparticle [21]. The AT1 peptide is an 8-amino acid peptide chain of Asp-Arg-Val-Tyr-Ile-His-Pro-Phe. On the N-terminal of the chain, 4 Gly residues were added to serve as a spacer to form NH_2_-Gly-Gly-Gly-Gly-Asp-Arg-Val-Tyr-Ile-His-Pro-Phe-NH_2_ as given in a study by Dvir et al. [16]. The synthesis of the AT1-HSA was through a two-step chemical reaction using heterobifunctional cross-linkers. In the first step, the primary amines present on the surface of HSA molecules react with the amines on PA-(PEG)_4_-SPA. In the second step, EDC (pH 5.5) and Sulfo-NHS were added followed by addition of the AT1 peptide. The EDC reacts with the carboxyl group on one end and Sulfo-NHS reacts with amine present on the AT1 peptide, releasing the unstable intermediates and forming AT1-HSA (Figure 1).

### 2.2. Nuclear Magnetic Resonance

**Figure 2:**
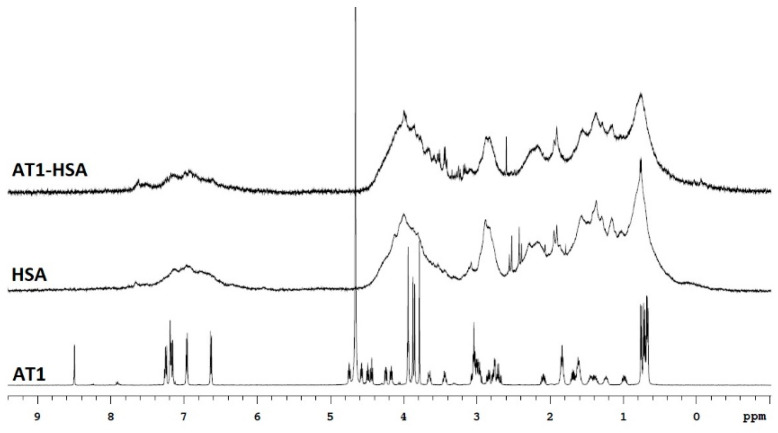
1H NMR-based characterization of AT1-HSA, HSA, and AT1 peptide. The AT1 peptide exhibits a spectrum with sharp peaks at δ = 0.5–1.0, δ = 1.1–2.1, δ = 2.8–3.8, δ = 4.0–5.0, and δ = 6.3–7.3. The peaks at δ = 6.8 due to tyrosine and around δ = 3.8–4.0 due to glycine from the AT1 peptide can be seen on the AT1-HSA spectrum around δ = 3.5–4.0 and 6.8 ppm.

The binding of AT1 peptide with HSA was initially characterized by ^1^H-NMR (Figure 2). The NMR spectrum of AT1 displays peaks at 6.8–7.0 ppm which are due to the presence of tyrosine residues, while most of the downfield resonance is due to histidine [21]. Peaks at around 3.5–4.0 ppm due to glycine residues visible on AT1 spectrum are also visible on the AT1-HSA spectrum at around 3.5 ppm. However, due to the higher molecular weight of HSA (66500 Da) compared to the AT1 peptide (1274 Da), we observe broader peaks for HSA and AT1-HSA. Thus, the ^1^H-NMR indicates binding of the AT1 peptide with the HSA.

### 2.3. Mass Spectrometry

**Figure 3:**
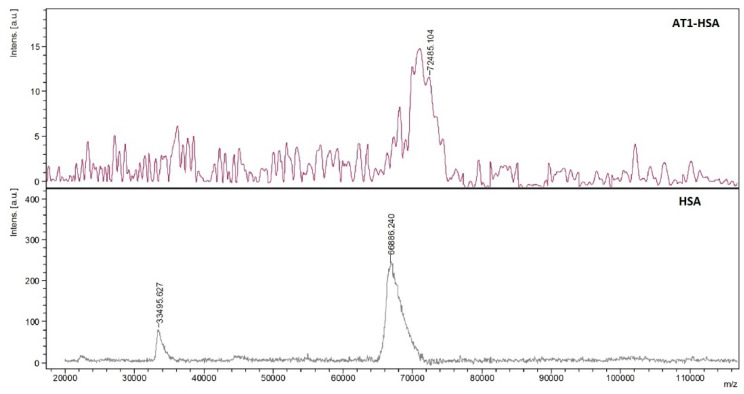
HSA (lower) and AT1-HSA (upper) were analyzed by Matrix-Assisted Laser Desorption/Ionization Time of Flight Mass Spectrometry. The m/z ratio of the AT1-HSA peak was at least 5600 higher than that of the HSA peak, which demonstrated that AT1 was successfully conjugated to the surface of HSA.

To validate the conjugation of AT1 with HSA, MALDI-TOF-MS was performed to compare the average molecular weight change between HSA and AT1-HSA. The mass-to-charge ratio (*m*/*z*) of the green peak (AT1-HSA) was approximately 5600 higher than the red peak (HSA) (Figure 3). Since the molecular weights of the AT1, PA-(PEG)_4_-SPA, EDC, and Sulfo-NHS are 1274, 435.4, 190, and 217 g/mol, respectively, it can be inferred that several molecules of the AT1 peptide are successfully conjugated to the surface of each HSA molecule.

### 2.4. Quantification of the AT1 Peptide

The quantity of AT1-peptide attached to the HSA molecule was determined by UV-visible spectrophotometry. Results showed that 82.9 ± 1.6% of the starting concentration of the AT1-peptide remained bound to the surface of HSA after the chemical conjugation reaction.

### 2.5. Characterization of the AT1-HSA-MRN-NPs

The size of the nanoparticles was determined by DLS and zeta potential was measured by laser Doppler anemometry. The particle size and zeta potential of AT1-nanoparticle formulation at MRN/AT1-HSA (*w*/*w*) ratio of 1:10 was 190.2 ± 5.7 nm with a zeta potential of −29.5 ± 3.7 mV. The size of the nanoparticles at MRN/AT1-HSA (*w/w*) ratio of 1:20 was 205.6 ± 3.8 nm with zeta potential of −27.5 ± 4.6 mV. The size of the nanoparticles at MRN/AT1-HSA (*w/w*) ratio of 1:40 was 225.4 ± 2.8 nm with zeta potential of −20.5 ± 4.4 mV. The size of the nanoparticles at MRN/AT1-HSA (*w/w*) ratio of 1:80 was 245.6 ± 3.5 nm with zeta potential of −18.7 ± 6.6 mV. These results suggest that as the quantity of MRN bound to the nanoparticles increased, there was reduction in the size of the nanoparticles and the zeta potential became more negative, indicating greater physical stability of the particles. The yield and drug encapsulation efficiency of the AT1-nanoparticles has been summarized in Table 1.

**Figure 4:**
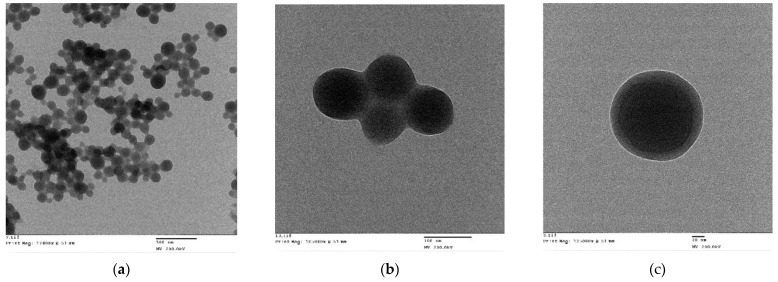
Nanoparticle surface characterization using TEM analysis: (**a**) Under magnification of 17,800×, AT1-HSA-MRN-NPs of size 190.2 ± 5.7 nm with a zeta potential of −29.5 ± 3.7 mV (Scale = 500 nm); (**b**) Under magnification of 105,000×, AT1-HSA-MRN-NPs with moderately uniform particle size (Scale = 100 nm); (**c**) Under 135,000× magnification, the AT1-HSA-MRN-NPs display a dark core surrounded by a bright distinct membrane layer (Scale = 20 nm).

The morphology of the nanoparticles observed by TEM under 17,800× (Figure 4a) and 105,000× magnification exhibited a near spherical shape with moderately uniform particle size and distribution (Figure 4b). Under 135,000× magnification, the AT1-HSA-MRN-NPs had a dark core surrounded by a bright membrane, which confirmed the distinct layer of peptide bound to the surface (Figure 4c).

The AT1-HSA-MRN-NPs were further tested in vivo for determining pharmacokinetics, biodistribution, safety and toxicity, and cardiac function assessment. In vitro studies on nanoparticle optimization, MRN binding and release, intracellular uptake, and biocompatibility have been studied extensively and reported through prior research and therefore were not repeated in this study [9,10].

### 2.6. Pharmacokinetic Parametersv

**Figure 5:**
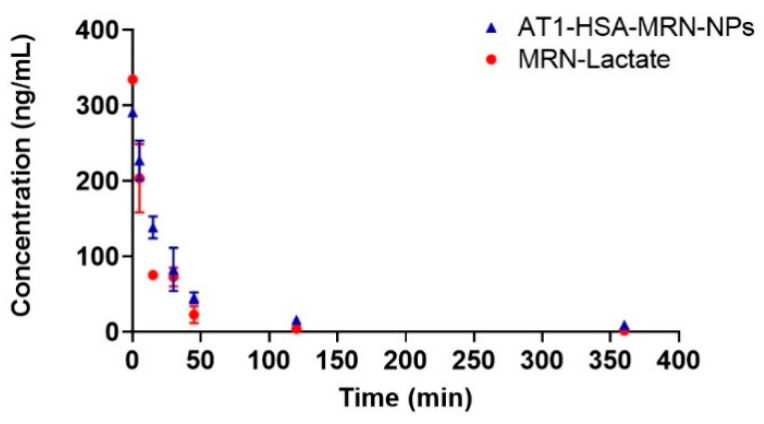
Pharmacokinetics of AT1-HSA-MRN-NPs (Group II) and MRN-Lactate (Group III) at an initial MRN dose of 50 μg/kg in vivo.

To determine MRN pharmacokinetics, female Lewis rats were divided into three groups. Group I received an injection of saline, Group II was injected with AT1-HSA-MRN-NPs at a milrinone dose of 50 μg/kg and Group III received MRN Lactate at a milrinone concentration of 50 μg/kg. The above dosage was determined from previous studies and keeping aligned with the dosage of milrinone lactate that is clinically administered to heart failure patients [22,23]. The milrinone plasma concentration-time curves of the intravenously administered formulations are represented in Figure 5. Results suggest that the MRN Lactate was removed from circulation much quicker than the AT1-HSA-MRN-NPs. The MRN Lactate concentration 6 h post injection was extremely low, however MRN released from the AT1-nanoparticles was detected even 6 h post injection. The clearance of the AT1-HSA-MRN-NPs was significantly delayed, as shown in Table 2 listing milrinone concentrations (ng/mL) at the various time points (0, 5, 15, 30, 45, 60, 120, and 360 min) for Group II injected with AT1-MRN-HSA-NPs and Group III injected with MRN Lactate, both intravenously.

The mean pharmacokinetic parameters were calculated using a non-compartmental method and have been listed in Table 3. For AT1-HSA-MRN-NPs, the MRT was 123.7 min, AUC was 183.9 ng*h/mL, half-life was 101.3 min, and clearance rate was 0.24 L/(kg*h). For MRN Lactate, the MRT was 49.1 min, AUC was 104.3 ng*h/mL, half-life was 64.7 min, and clearance rate was 0.47 L/(kg*h).

### 2.7. Tissue Distribution Study

**Figure 6:**
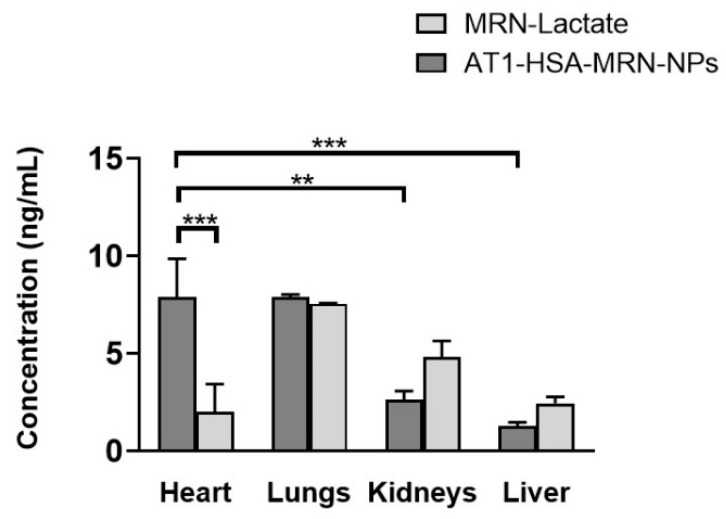
Tissue distribution of AT1-HSA-MRN-NPs and MRN-Lactate at an initial MRN dose of 50 μg/kg in vivo. The graph shows a representative result of mean ± SD (*n* = 4). *** *p* < 0.001 was considered highly significant and ** *p* < 0.01 was considered significant based on Tukey’s post hoc analysis.

The concentration of MRN in AT1-HSA-MRN-NPs and MRN Lactate was investigated after intravenous administration of the formulations in female Lewis rats. The amounts of MRN detected at 2 h time point in different tissues such as heart, lungs, kidneys, and liver are shown in Figure 6. The level of MRN uptake for the lungs, kidneys, and liver was greater in the MRN Lactate group (Group III) than in the AT1-HSA-MRN-NP group (Group II). For lungs, the MRN detected in the MRN Lactate group vs. AT1-HSA-MRN-NPs was 7.55 ± 0.01 vs. 7.88 ± 0.09 ng/mL, for kidneys was 4.80 ± 0.6 vs. 2.64 ± 0.30 ng/mL, and for liver was 2.44 ± 0.23 vs. 1.27 ± 0.14 ng/mL. This is due to the passive targeting of albumin nanoparticles. Interestingly, the amount of MRN detected in the heart was significantly higher (*p* < 0.01) in case of the AT1-HSA-MRN-NPs group (7.88 ± 1.4 ng/mL) vs. the MRN-Lactate (2.00 ± 1.01 ng/mL). The MRN uptake by the heart was significantly greater than the MRN detected in other tissues for Group II, which indicates the target specificity of the nanoparticles towards the AT1 receptors present on the myocardium. For the same group, there was no significant difference in MRN uptake between the heart and lungs.

### 2.8. Cardiac Function Assessment

**Figure 7:**
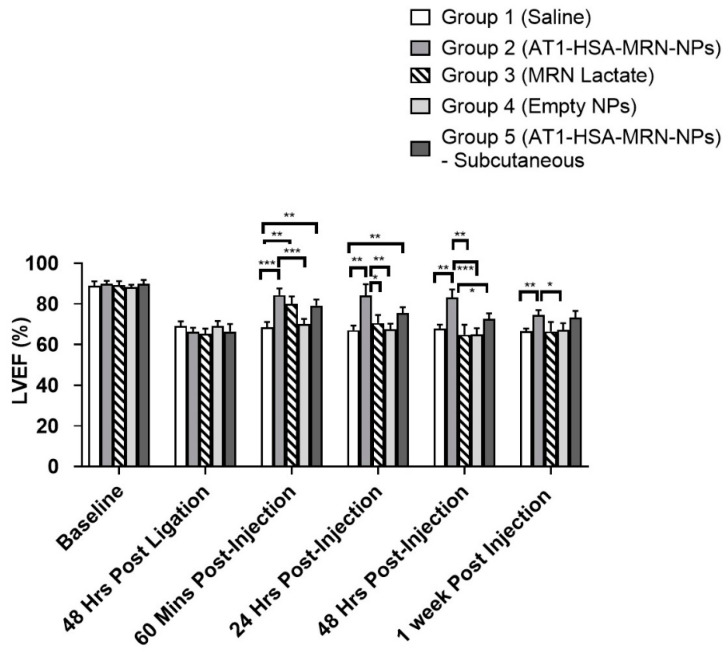
Percentage LVEF measurements for Groups 1, 2, 3, 4, and 5 at baseline, 48 h post ligation, 60 min, 24 h, 48 h, and 1-week post injections. The graph shows a representative result of mean ± SD (*n* = 5). *** *p* < 0.001, ** *p* < 0.01 and * *p* < 0.05 were considered significant based on Tukey’s post hoc analysis.

The echocardiographic parameters were analyzed by comparing the baseline values with the subsequent echocardiographic measurements taken post surgery and injection (Figure 7). The percentage left ventricular ejection fraction (LVEF %) and percentage left ventricular fractional shortening (LVFS %) were measured for all the groups at pre-operation/ligation, 48 h post ligation, 60 min post treatment injections, 24 h post injections, 48 h post injections and 1-week post injection (Table 4 and Table 5). The EF was not significantly different between the groups preoperatively (baseline) and 48 h post ligation. A two-way ANOVA determined that the EF at 60 min post injection for Group II (84.3 ± 2.9%) was higher than Group 1 (68.6 ± 2.2%) (*p* = 0.0003) and Group 4 (70.1 ± 2.3%) (*p* = 0.0006) however, not significantly different than Groups 3 (*p* = 0.3846) and 5 (p = 0.1744). At 24 h post injection, the EF for Group 2 remained consistent until 1-week post ligation. At 24 h post treatment, there was a reduction in EF for Group 3 (70.6 ± 3.6%), which was significantly lower (*p* = 0.0152) than Group 2 (84.2 ± 4.8%) in addition to Groups 1 and 4. This effect stayed consistent at 48 h post injection with a reduction in Group 5 (72.7 ± 2.3%) EF as well, which was lower (*p* = 0.0114) than that of Group 2 (83.1 ± 3.6%). This indicates that the intravenous (i.v.) delivery route worked better for targeted drug delivery as compared to the subcutaneous route of delivery in terms of sustained drug release. At 1-week post injections, there was no significant differences between Groups 2, 3, and 5, however EF of Groups 2 was higher than Group 1 (*p* = 0.0038) and 4 (*p* = 0.0259).

**Figure 8:**
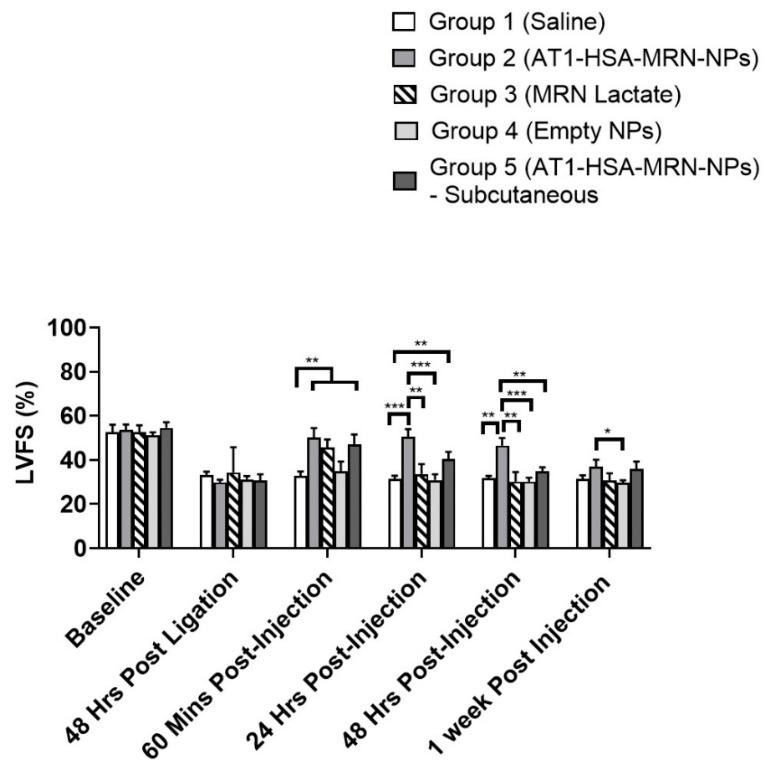
Percentage LVFS measurements for Groups 1, 2, 3, 4, and 5 at baseline, 48 h post ligation, 60 min, 24 h, 48 h, and 1-week post injections. The data have been presented as mean ± SD (*n* = 5). *** *p* < 0.001 was considered highly significant and ** *p* < 0.01, * *p* < 0.05 was considered significant based on Tuckey’s post hoc analysis.

Similarly, for FS measurements, there was no significant differences amongst the groups either pre-ligation or 48 h post ligation (Figure 8). However, in accordance with EF measurements, the FS for Group 2 (50.1 ± 3.8%) was significantly higher than that of Groups 1 (32.8 ± 1.7%) (*p* = 0.0016) and 4 (34.7 ± 4.0%) (*p* = 0.0037) at 60 min post injection, with no significant difference from Groups 3 and 5. The FS of Group 3 was reduced at 24 h (33.6 ± 3.9%) and 48 h (30.2 ± 3.9%) post injection, which was significantly lower (*p* = 0.0014) than that of Group 2 at those time points (50.4 ± 3.1% and 46.5 ± 3.1%, respectively). A similar effect was observed for Group 5 as well in which the FS was reduced significantly in comparison to Group 2 at 24 h (40.5 ± 2.8%) (*p* = 0.0107) and 48 h (34.8 ± 1.7%,) (*p* = 0.0029). At 1-week post injections, there was reduction in FS of Group 2 with no significant differences amongst the groups, except with Group 4 (*p* = 0.0182).

### 2.9. Milrinone Quantification from Rat Plasma

**Figure 9:**
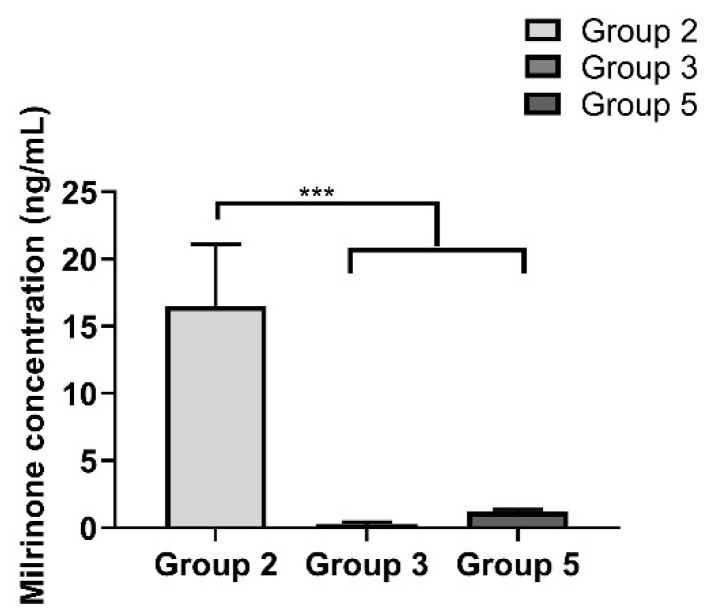
Milrinone levels detected in plasma 24 h post treatment for Groups 2, 3, and 5. The graph shows a representative result of mean ± SD (*n* = 3). *** *p* < 0.001 was considered significant based on Tukey’s post hoc analysis.

Both milrinone and amrinone were detected by LC-MS. Results showed that at 24 h post treatment, the milrinone detected in plasma in Group 2 was significantly greater than in Groups 3 and 5 (Group 2: 16.5 ± 4.6 ng/mL vs. Group 3: 0.3 ± 0.1 ng/mL and Group 5: 1.2 ± 0.1 ng/mL) (Figure 9).

### 2.10. Safety and Toxicity of the AT1-HSA-MRN-NPs

**Figure 10:**
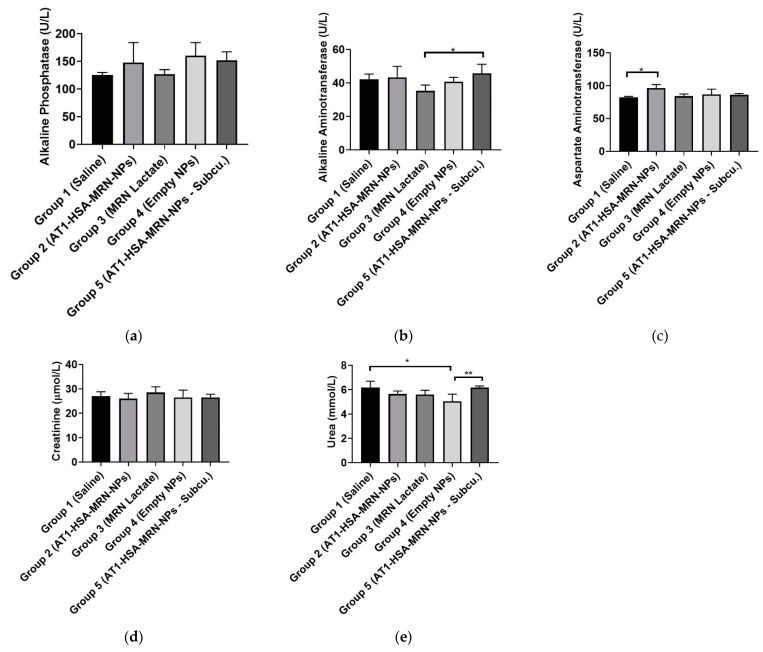
Serum analysis performed as a safety test comparing the (**a**) ALP (U/L); (**b**) ALT (U/L); (**c**) AST (U/L) as liver function tests and (**d**) creatinine (μmol/L); (**e**) urea (mmol/L) as kidney function tests. The graph shows a representative result of mean ± SD (*n* = 5). ** *p* < 0.01, * *p* < 0.05 were considered significant based on Tukey’s post hoc analysis.

The serum samples collected from the animals were analyzed for markers of safety and toxicity. The liver function and toxicity analysis were performed by analyzing alkaline phosphatase (ALP), aspartate aminotransferase (AST), and alkaline aminotransferase (ALT), respectively (Figure 10a–c). Results show that there were no significant differences in the ALP levels between the groups and all values fall in the normal range of 16–302 U/L for rats. The levels of ALT enzyme for Group 3 were lower than Group 5 (*p* = 0.0350), but were within the normal range of 20–61 U/L. The AST enzyme levels of Group 2 were elevated as compared to Group 1 (*p* = 0.0273), however, were well within the normal range of 39–111 U/L and did not differ significantly between the control and other treatment groups. The GGT levels were also measured and were found to be in range (0–6 U/L) for all groups. To evaluate the renal function, creatinine and urea were analyzed (Figure 10d–e). It was found that though there was no significant difference in the creatinine levels amongst the groups, these values were lower than the normal range of 50–73 μmol/L. This is indicative of renal dysfunction which is commonly associated with heart disease. There were significant differences in urea levels between the Groups 1 and 4 (*p* = 0.0096) and Groups 4 and 5 (*p* = 0.0096), however the levels were found to be in the normal range overall (3.2–7.5 mmol/L).

### 2.11. Serum Cytokine Measurements

**Figure 11:**
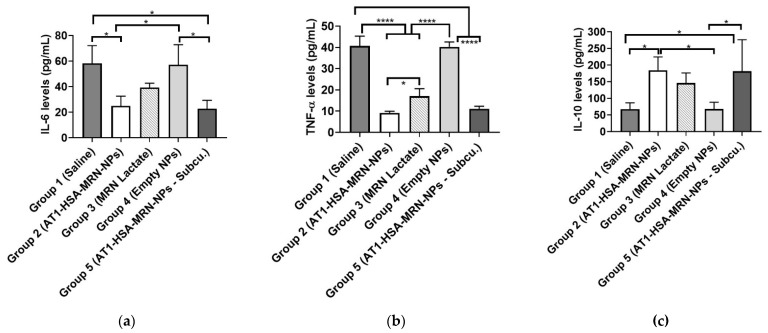
Serum cytokine levels for (**a**) IL-6 (pg/mL), (**b**) TNF-α (pg/mL), and (**c**) IL-10 (pg/mL). Groups 2 and 5 had significantly lower levels of IL-6 and TNF-α and significantly higher levels of IL-10 compared with other groups. The data have been presented as mean ± SD (*n* = 5). **** *p <* 0.0001 was considered highly significant and *p < 0.05 was considered significant based on Tukey’s post hoc analysis.

The serum cytokine levels of IL-6, IL-10, and TNF-α were measured at 1-week post treatment (Figure 11). Serum TNF-α levels were significantly different amongst the groups with control Groups 1 at 40.7 ± 3.8 pg/mL and Group 4 at 40.3 ± 1.8 pg/mL, which was higher (*p* < 0.0001) than that of Group 2 (9.2 ± 0.6 pg/mL), Group 3 (17.1 ± 2.8 pg/mL, and Group 5 (11.1 ± 0.9 pg/mL) (Figure). The Group 2 and Group 5 TNF-α levels were significantly higher (*p* < 0.05) than the serum TNF- α levels of Group 3 (*p* = 0.0442), with no significant difference between Groups 2 and 5. Serum IL-6 levels were significantly different between the groups. Group 1 and Group 4 IL-6 levels (58.2 ± 11.3 pg/mL and 57.1 ± 12.8 pg/mL) were significantly higher (*p* < 0.05) than that of Group 2 (24.9 ± 6.2 pg/mL), Group 3 (39.3 ± 2.7 pg/mL), and Group 5 (22.6 ± 5.4 pg/mL) (*p* = 0.0137). There were no significant differences between Groups 1 and 4 and between Groups 2, 3, and 5. However, the anti-inflammatory cytokine IL-10 levels in Groups 1 (67.3 ± 15.5 pg/mL) and 4 (71.1 ± 12.1 pg/mL) were significantly lower (*p* < 0.05) than Group 2 (184.4 ± 32.7 pg/mL), Group 3 (146.1 ± 24.7 pg/mL), and Group 5 (181.6 ± 77.3 pg/mL).

## 3. Discussion

Nanoparticle-based technology has advanced significantly in the last few decades with numerous applications in the field of medicine and healthcare. Nanoparticle-based drug delivery systems have shown innovative approaches of loading and delivering drugs, genes, hormones, small molecules, etc., in both targeted and non-targeted ways [12,24]. This allows for targeted and site-specific delivery, controlled drug release, enhanced bioavailability, improved safety, and reduced toxicity [5,6,25]. Though most of these formulations have been tested and approved for use in cancer, their use in cardiovascular applications such as congestive heart failure is equally promising with maximum focus on in-stent restenosis and the use of microparticles for drug delivery [26].

In the present study, AT1-HSA-MRN-NPs have been successfully prepared as a novel heart-targeted formulation for site-specific delivery of MRN using albumin nanoparticles, surface functionalized with the ligand, AT1 peptide. Under conditions of MI and HF, AT1 receptors, present on the myocardium, are found to be overexpressed [8,17,27]. Thus, the AT1 receptors, specific for the angiotensin II peptide, facilitate uptake of the AT1-HSA-MRN-NPs through receptor-mediated endocytosis allowing for site-specific unloading of the MRN drug. The albumin protein is a heterodimer with sites for binding many hydrophobic and hydrophilic molecules along with active functional groups such as amino and carboxylic groups for covalent conjugation [28,29]. Exploiting these properties, the HSA molecule was first surface-modified to bind the AT1 ligand using PA-(PEG)_4_-SPA, EDC, and Sulfo-NHS as crosslinkers. This was confirmed through NMR and mass spectrometry which indicated that AT1 was bound to the HSA molecule. UV-visible spectrophotometry analysis further revealed that approximately 83% of the AT1 peptide remained bound to albumin post the covalent chemical conjugation treatment and purification.

The newly synthesized AT1-HSA was used to bind MRN and prepare AT1-HSA-MRN-NPs of particle size 190.2 ± 5.7 nm and zeta potential of −29.5 ± 3.7 mV. It is known that the optimal nanoparticle size to avoid rapid clearance from the body by the RES and macrophages is up to 250 nm with a highly positive or highly negative surface charge [30,31]. The particles developed in this study were reproducible with the intended nanoparticle diameter. Through our previous studies, we have successfully confirmed the binding and release of MRN from the nanoparticles as well as the in vitro safety and intracellular uptake characteristics [10]. Hence, these studies were not repeated and instead focused on in vivo evaluation. 

Typically, post intravenous injections, a controlled and sustained drug release is desired as opposed to a burst release effect. A sustained nanoparticle-drug release eliminates the requirement of a continuous drug infusion or multiple drug injections for treatment. The pharmacokinetics of the AT1-HSA-MRN-NPs and MRN Lactate were studied in a rat model at MRN dose of 50 μg/kg in a 0.25 mL i.v. injection at 0, 5, 15, 30, 45, 120, and 360 min. Compared to MRN Lactate, the AT1-HSA-MRN-NPs showed a higher AUC and prolonged residence of the MRN in blood, exhibiting the sustained release desired. Further, the higher accumulation of the drug in the heart at 2 h demonstrated that the nanoparticles could be targeted to the heart and reduce the side effects of the drug to other organs. Clinically, heart failure patients are given MRN Lactate at a milrinone concentration of 50 μg/kg [23]. Hence, keeping in alignment with the above dosage and previous studies, the above dosage was used for this study [22]. Studying other higher or lower doses was beyond the scope of this work and will be studied in the future. When MRN Lactate is intravenously injected, the direct interaction of the drug results in its fast elimination, however, loading the drug onto nanoparticles prevents exposure to blood components, acting as a reservoir for a maintaining a controlled release over time. Thus, it may be concluded that the nanoparticles improve the delivery of MRN with prolonged drug retention and targeted uptake by the tissue, in vivo.

Milrinone has been widely used for the treatment congestive heart failure resulting in a low cardiac output, right ventricular failure, and pulmonary tension [23,32]. For patients with end stage heart failure, awaiting heart transplantation or ventricular assist devices, milrinone is used as a long-term continuous infusion [33,34]. In this study, the recovery of heart function was evaluated by measuring both LVEF and LVFS. Both LVEF and LVFS first decreased in response to the infarction created for 48 h. On injecting the formulations, the LVEF increased significantly for Groups 2, 3 and Group 5, when observed at 60 min post treatment, with no significant difference in Groups 1 and 4. There was no significant change in the LVEF of Group 2 until 48 h post treatment, however, the effect of MRN Lactate began to decrease with a reduction in LVEF over 48 h and 1 week. There were no significant variations between Group 2 and 5. Thus, the subcutaneous route of delivery did not show any difference from the intravenous delivery route. There was a similar trend in the LVFS parameter as well. It can be concluded that the AT1-HSA-MRN-NPs helped improve the cardiac function post heart failure. These results were in alignment with a similar study done using microparticles containing MRN Lactate [22]. In fact, in the current study, the effect of the sustained MRN release from the nanoparticles was visible for up to 7 days post treatment, indicated by the ejection fraction and fractional shortening. Though the milrinone dose of 50 μg/kg was consistent with previous studies, it is anticipated that injecting a higher dose of MRN will improve the drug release behaviour and deliver a larger quantity of drug in lesser time, without a cytotoxic effect.

Previous studies were mainly focused on determining the tissue levels of cytokines post infarction and not the serum levels. Hence, the cytokine levels in serum were determined with a focus on IL-6, IL-10, and TNF-α. The IL-6 and TNF-α levels were elevated in the infarcted myocardium as a result of remodelling, whereas the IL-10 levels were reduced. The groups treated with AT1-HSA-MRN-NPs had significantly reduced IL-6 and TNF-α, whereas the cardioprotective anti-inflammatory cytokine IL-10 was higher. Though, it is usual to observe higher pro-inflammatory cytokine levels even 1-week post infarction [13]. So, a beneficial effect of the novel nanoparticle formulation could be observed with the reduction in pro-inflammatory cytokines and elevation of the anti-inflammatory cytokines.

It can be concluded that this novel targeted nanoparticle formulation presents a new approach towards the treatment of congestive heart failure. This is the first study to deliver a cardiac inotropic drug in a targeted manner using biodegradable nanoparticles, displaying the effectiveness of the nanoparticle formulation in comparison with the free drug. Though, the nanoparticle formulation was delivered intravenously and subcutaneously in this study, an intranasal route of delivery could also be evaluated as a non-invasive procedure for future work. To accomplish the same, further optimization of the nanoparticle formulation will be needed to achieve a particle size of less than 100 nm to prevent capillary clogging. Another limitation of this study is that any potential side effects associated with the use of MRN, such as arrhythmias, have not been studied in comparison to the nanoparticle formulation, when tested in vivo. This can be addressed by studying toxicity and cardiac function at various MRN doses. This study must be tested in male rats as well as female rats to study the impact of gender differences in the cardiovascular function and pharmacokinetics. The biodistribution study must be performed in organs other than heart, liver, kidney, and lungs, to understand passive targeting of the targeted nanoparticle formulation. Lastly, a large scale and long-term study is required to study the overall treatment efficacy of the targeted nanoparticle formulation and evaluate the improvement in cardiac function upon its use.

Though in the current study, targeted albumin nanoparticles were synthesized to deliver a drug, it is anticipated that the effect of this treatment could be further enhanced by using this nanoparticle system to also deliver genes or growth factors in addition to drugs, promoting myocardial regeneration. Additionally, it has been reported that the phosphodiesterase-3 varies across different species. Thus, investigating the effects of this novel nanoparticle formulation would be useful in developing a comprehensive understanding of the species variation.

## 4. Materials and Methods

### 4.1. Materials

Human serum albumin (>97% lyophilized) was purchased from Sigma Aldrich (Oakville, ON, Canada). Glutaraldehyde (25% aq. solution) was purchased from Alfa Aesar (Cedarlane, Burlington, ON, Canada). Milrinone was purchased from Selleck Chemicals (Burlington, ON, Canada). EDC, Sulfo-NHS were purchased from Thermo Fisher Scientific (Missisauga, ON, Canada) Other chemicals were purchased from Fisher Scientific (Nepean, ON, Canada).

### 4.2. Synthesis of the AT1 Peptide

The Angiotensin II Type 1 (AT1) receptor targeting peptide is a chain of eight amino acids Asp-Arg-Val-Tyr-Ile-His-Pro-Phe, and was synthesized by CanPeptide (Pointe-Claire, QC, Canada) as NH_2_-Gly-Gly-Gly-Gly-Asp-Arg-Val-Tyr-Ile-His-Pro-Phe-NH_2_ following the sequence mentioned by Dvir et al. [16].

### 4.3. Surface Modification of HSA with AT1 Peptide

The surface of HSA was modified for attachment of the AT1 peptide in a two-step reaction. An aqueous solution of 20 mg/mL of HSA dissolved in deionized water (0.3 mM) was prepared and reacted with PA-(PEG)_4_-SPA (10-fold molar excess), for 1 h. The solution was further mixed with EDC/Sufo-NHS for 30 min followed by reaction with the AT1 peptide (10-fold molar excess) for 4 h. The AT1-HSA was purified by dialysis using the Slide-a-Lyzer dialysis cassette (10K Da MWCO). The purified sample was lyophilized and stored at 4 °C.

### 4.4. Nuclear Magnetic Resonance

The 1D proton spectra were recorded at 25° on a 500 MHz Varian INOVA NMR Spectrometer, with an HCN triple resonance RT probe with *Z*-axis pulsed field gradients. Spectra were recorded with double pulsed-field gradient spin echo for suppression of residual H_2_O signal. Further, 256 scans with a recycle delay of 1 s were collected with a sweep width of 8000 Hz and an acquisition time of 1 s. Data were processed with 1 Hz line broadening using the VNMRJ 4.2 software. The concentrations of HSA, AT1 peptide, and AT1-HSA were 5 mg/mL in D_2_O.

### 4.5. Mass Spectrometry

The AT1-HSA, AT1 peptide, and HSA samples were analyzed by the Matrix-Assisted Laser Desorption/Ionization Time of Flight Mass Spectrometry (MALDI-TOF-MS) MALDI Autoflex III- TOF-(BRUKER) SMARTBEAM) (Dept. of Chemistry, McGill University, Montreal, QC, Canada) in linear positive mode. Dihydroxybenzoic Acid was used as the Matrix and the AT1-HSA, HSA, and AT1 peptide samples were dissolved in water at concentrations of 7 mg/mL

### 4.6. Quantification of AT1 Peptide Attached to HSA

The amount of AT1 peptide attached to the HSA molecule was determined by UV-Visible spectrophotometry. Post surface modification of HSA with AT1 by chemical conjugation and prior to dialysis, the reaction mixture was centrifuged using Amicon centrifugal filters with 30 KDa MWCO. The unbound AT1 peptide (Mol. Wt. 1292 g/mol) was collected as the filtrate at the bottom of the tube and quantified by UV-Visible spectrophotometry at 280 nm. A standard curve was prepared by making serial dilutions of AT1 to measure the unknown quantity of AT1 in solution.

### 4.7. Nanoparticle Preparation

The AT1-HSA-MRN-NPs were prepared by the ethanol desolvation technique [35,36]. Briefly, an aqueous solution AT1-HSA (20 mg/mL) was prepared in deionized water and solution pH was adjusted to pH 8.0 using 0.1 M NaOH. MRN was dissolved in DMSO and added to the AT1-HSA, with MRN/AT1-HSA (wt./wt.) at 1:10, 1:20, 1:40, and 1:80 [10]. Ethanol was added in a dropwise manner resulting in solution turbidity. Glutaraldehyde (8% *v/v* aq. solution) was added at a concentration of 0.588 μL/mg HSA and reacted for 24 h. The nanoparticles were washed by three rounds of ultracentrifugation at 18,000× *g* for 15 min each at 25 °C. The supernatant was collected for detection of unbound MRN. The pellet was washed with deionized water and resuspended in PBS.

### 4.8. Nanoparticle Characterization

The average size of the nanoparticles was measured by Dynamic Light Scattering (DLS) using a Particle Size Analyzer (Brookhavens Instruments Corporation, Holtsville, NY, USA). The samples were diluted 1:20 with deionized water and measured at a scattering angle of 90⁰ and temperature of 25 °C. The Polydispersity Index (PDI) estimated the size distribution of the nanoparticles. The zeta potential was measured by a Zeta Potential Analyzer (Brookhavens Instruments Corporation, Holtsville, NY, USA) using electrophoretic laser Doppler anemometry. The size, shape, and surface morphology of the nanoparticles were examined by SEM and TEM techniques.

The yield of the nanoparticles was measured by the UV-spectrophotometric method [9,10]. A standard curve of HSA solution dissolved in Bradford reagent was used as a reference and absorbance was measured at 595 nm. For measuring encapsulation efficiency, nanoparticles were spin concentrated using centrifugal filters (10K Da MWCO) for eluting the non-encapsulated MRN into the collection tube. The concentration of non-encapsulated MRN was determined by UV-spectrophotometry at 356 nm. A standard curve of MRN in a DDQ/Ethanol mixture was used as reference [37].

### 4.9. In Vivo Studies

All experiments were performed on female Lewis Rats (200–250 g; Charles River Laboratories, Senneville, Quebec Canada) in accordance with the guidelines set forth by the Canadian Council on Animal Care and were approved by the institutional ethics committee. Rats were housed in groups of two to three per cage.

Two in vivo studies were performed. The first study includes pharmacokinetics and tissue distribution analyses on rats not having undergone the coronary artery ligation surgery. The second study evaluated the treatment efficacy of the targeted nanoparticle formulation in a rat model of CHF by performing ligation surgery of the left anterior descending (LAD) coronary artery.

### 4.10. Pharmacokinetics and Biodistribution Study

The animals were randomized into three groups in a blinded manner. Group I (*n* = 6) was intravenously injected with 1 c.c. saline. Group II (*n* = 12) was intravenously injected with 250 μL of AT1-HSA-MRN-NPs containing 50 μg/kg MRN. Group III (*n* = 12) was intravenously injected with 250 μL of MRN Lactate containing 50 μg/kg MRN. Blood was collected from the jugular vein at the following timepoints: 0, 5, 15, 30, 45, 60, 120, and 360 min. The animals were euthanized by isoflurane/CO_2_. Organ tissues (heart, lungs, liver, and kidneys) were collected, washed, rinsed, and snap-frozen in liquid nitrogen.

### 4.11. Ligation of the Left Anterior Descending Coronary Artery to Induce CHF

The surgery was performed in a blinded manner as previously mentioned [22,38]. Briefly, rats were anesthetized using 5% isoflurane, intubated, and mechanically ventilated at 80 breaths/minute. Via a left thoracotomy (through the fourth intercostal space), the LAD coronary artery was permanently ligated 2 mm from its origin with a 7/0 polypropylene suture (Ethicon Inc., Somerville, NJ, USA). The ischemic myocardial segment rapidly became identifiable through its pallor and akinesia corresponding to the distribution of the LAD coronary artery territory distal to the occlusion, which resulted in MI of the free left ventricle (LV) and subsequently heart failure. About 48 h after ligation of the artery, injections were performed using a 27-G needle. Rats were randomized into five groups: Group 1 (*n* = 5) received an intravenous injection of 1 c.c. saline (Control). Group 2 (*n* = 8) received 250 μL intravenous injection of AT1-HSA-MRN-NPs containing 50 μg/kg of milrinone. Group 3 (*n* = 8) received 250 μL bolus intravenous injection of 50 μg/kg MRN Lactate [39,40]. Group 4 (*n* = 5) received 250 μL intravenous injection of empty NPs in the same concentration as Group 1. Group 5 (*n* = 8) received 250 μL subcutaneous injection of 50 μg/kg of AT1-HSA-MRN-NPs. The reason for using empty NPs as a control was to ensure that these particles had no effect on cardiac function or other inadvertent toxicity. Blood was collected 24 h post injection for milrinone quantification. Various endpoint measurements were taken as described in subsequent paragraphs. At 5 days post injection, all rats were killed by euthanasia. The hearts were washed with saline solution to remove excess blood and clots and then fixed in neutral-buffered 4% formalin.

### 4.12. Animal Mortality

Forty female Lewis rats were included in the study, with a total of thirty-four rats surviving till the 1-week experimental end point of the study. All mortalities occurred during surgery. There was no difference in mortality among the different groups. No mortality was observed in surviving rats.

### 4.13. Blood Serum and Plasma Collection

Blood was collected via the jugular vein from the animals using a sterile 23 G/25 mm needle in the Microtainer® serum separator tubes (Becton Dickinson, Franklin Lakes, NJ, USA) for serum separation and in Plasma-EDTA tubes (Becton Dickinson, Franklin Lakes, NJ, USA) for plasma collection. The blood was allowed to clot at room temperature for 30 min and subsequently placed on ice until centrifugation. Serum was separated by low-speed centrifugation at 1500 rpm for 15 min at 4 °C and was frozen at −80 °C until analysis. Serum was used to test for C-reactive protein (CRP) and liver function tests, alkaline phosphatase (ALP), alanine aminotransferase (ALT), and aspartate transaminase (AST). Urea, creatinine (CRE), and uric acid (UA) were also tested for renal functionality in the animals using a conventional enzymatic method on Hitachi 911 automated clinical chemistry auto-analyzer (Roche Diagnostics, Indianapolis, IN, USA). The plasma was separated by low-speed centrifugation at 2500 rpm for 10 min at 4 °C and was frozen at −80 °C until analysis. Plasma was used to detect milrinone levels using High Performance Liquid Chromatography (HPLC).

### 4.14. Quantification of Milrinone from Rat Plasma

The milrinone in plasma samples was quantified using High Performance Liquid Chromatography (HPLC) LCMS-8060 (Shimadzu). For sample preparation, stock solutions of milrinone (1 mg/mL) and amrinone (1 mg/mL) were prepared in DMSO. Amrinone was used as an internal standard. A working solution of amrinone at 250 ng/mL was prepared by serial dilution with deionized water. Further, 50 μL of plasma was mixed with 10 μL of internal standard and 500 μL of ethyl acetate was added. The sample was vortexed and centrifuged at 15,000× *g* for 5 min and 400 μL supernatant was separated. A standard curve was generated using rat plasma that was spiked with 1000 ng/mL milrinone and serially diluted. The standard curve samples were then spiked with 10 µL of internal standard working solution (0.25 ng/mL amrinone) and extracted with 500 µL of ethyl acetate and 400 µL of supernatant was dried. All samples were reconstituted in 40 µL of mobile phase A and analyzed by LC-MS/MS. Separation was performed on a reversed phase Kinetex XB 50 mm × 2.1 mm column which was maintained at 40 °C. Samples were injected by loop overfilling into a 5 μL loop. For the 6 min LC gradient, the mobile phase consisted of the following solvent A (5 mM ammonium formate at pH 4.5 in water) and solvent B (5 mM ammonium formate in methanol) at a flow rate of 400 μL/min. The gradient started at 95:5 A:B. Samples were quantified using the area under the curve (AUC) and a standard curve. Samples were integrated using the Browser extension from the Labsolution suite.

### 4.15. Echocardiography Procedure

Echocardiographic examinations were performed under inhaled isoflurane anesthesia (2.5% in oxygen, 500–700 mL/min). Transthoracic echocardiography was performed for each rat as a baseline before the surgery, 48 h post surgery, 60 min post injection, 24 h post injection, 48 h post injection, and 1-week post injection. Echocardiograms were obtained with a commercially available system (Micromaxx P04224; SonoSite, Bothell, WA, USA), equipped with a linear probe 7–13 MHz 25 mm footprint turbo transducer (P06519.11; SonoSite). Briefly, LV end-diastolic diameters (LVEDD) and end-systolic diameters (LVESD) were measured with M-mode tracings between the anterior and posterior walls from the parasternal short-axis view just below the level of the papillary muscles of the mitral valve [13]. The time of end-diastole was defined as time of maximum diameter of the LV in one heart cycle. Accordingly, end-systole was defined as the minimum diameter. Following the American Society of Echocardiology leading-edge method, two images on average were obtained in each view and averaged over three consecutive cycles [41]. The left-ventricular end-diastolic volume (LVEDV) and left-ventricular end-systolic volume (LVESV) were measured using the Teichholz formula [13].

### 4.16. Cytokine Measurement

The cytokine analysis for levels of IL-6, IL-10, and tumor necrosis factor-alpha (TNF-α) was performed using commercially available ELISA kits (Abcam) according to the manufacturer’s instructions. Microtiter plates were precoated with a murine monoclonal antibody against the rat cytokine being measured. Standards of the analyte and serum samples were added in triplicate. The absorbance was read at 450 nm within 30 min of stopping the reaction and standard curves were plotted.

### 4.17. Tissue Collection and Histological Analysis

After 1 week of treatment, the animals were anesthetized and sacrificed using pentobarbital sodium (100 mg/kg) overdose. The heart, lungs, liver, and kidney were excised rapidly and washed with cold saline to remove excess blood and then fixed in neutral-buffered 10% formalin for 48 h. The tissues were trimmed to 3 mm thickness and stored in 70% ethanol in histology cassettes. The tissues were paraffin-embedded and processed into 4–5 μm thick sections on slides (The Rosalind and Morrison Goodman Cancer Research Institute, McGill University). The tissue slides were stained with hematoxylin-eosin following manufacturer’s instructions and observed by confocal microscopy at 400×.

## 5. Conclusions

A novel nanoparticle formulation has been developed for targeted MRN delivery. The HSA was surface-modified to form AT1-HSA, which was used to develop the AT1-HSA-MRN-NPs. As compared to the non-targeted MRN Lactate, the AT1-HSA-MRN-NPs exhibited prolonged drug release and superior pharmacokinetics and tissue distribution of MRN in vivo, improving cardiac function recovery with lesser toxicity and better pro-inflammatory/anti-inflammatory serum cytokine levels (IL-6, TNF-α, and IL-10) in a rat model of congestive heart failure. Thus, this presents a new strategy of delivering hemodynamically stable drugs in a prolonged manner with a sustained release profile with negligible side effects. It also exhibits the feasibility and effectiveness of nanoparticle-based formulations in cardiovascular medicine. This novel nanoparticle formulation could be used as a potent, safe, and non-toxic heart failure medication, with potential for use in other cardiovascular diseases.

## Figures and Tables

**Table 1:** Nanoparticle size, charge, encapsulation efficiency, and yield at various MRN/AT1-HSA (*w/w*) ratios of nanoparticle preparation.

MRN/AT1-HSA (*w/w*)	Nanoparticle Size (nm)	Zeta Potential (mV)	Encapsulation Efficiency (%)	Yield (%)
1:10	190.2 ± 5.7	−29.5 ± 3.7	41.8 ± 2.2	77 ± 2.3
1:20	205.6 ± 3.8	−27.5 ± 4.6	40.4 ± 1.5	75 ± 2.6
1:40	225.4 ± 2.8	−20.5 ± 4.4	54.1 ± 0.7	70 ± 3.5
1:80	245.6 ± 3.5	−18.7 ± 6.6	83.2 ± 1.2	68 ± 4.6

**Table 2:** Comparing milrinone concentration (ng/mL) at various time points between AT1-MRN-HSA-NPs (Group II) and MRN Lactate (Group III) injected intravenously in female Lewis rats. Table shows a representative result of mean ± SD (*n* = 4).

Time (min.)	AT1-HSA-MRN-NPs (ng/mL)	MRN Lactate (ng/mL)
0	291.2	334.2
5	227.3 ± 26.2	203.5 ± 45.3
15	138.5 ± 14.5	75.4 ± 6.2
30	82.6 ± 28.5	72.6 ± 12.4
45	45.3 ± 7.1	22.9 ± 11.2
120	15.9 ± 3.5	4.1 ± 1.5
360	9.1 ± 0.9	1.6 ± 0.1

**Table 3:** Pharmacokinetic parameters of AT1-HSA-MRN-NPs and MRN Lactate at MRN dose 50 μg/kg. C_0_: concentration at time 0; C_max_: maximum concentration; t_1/2_: half-life of plasma; MRT: mean residence time, AUC: area under the curve, Vz: apparent volume of distribution at elimination; Vss: apparent volume of distribution at steady state; CL: clearance.

Pharmacokinetic Parameters	MRN Dose: 50 μg/kg	
	MRN-Lactate	AT1-HSA-MRN-NPs
C_0_ (ng/mL)	203.5	291.2
C_max_ (ng/mL)	334.3	227.3
t_1/2_ (min)	64.7	101.3
MRT (min)	49.1	123.7
AUC_0-t_ (ng*h/mL)	104.3	183.9
AUC_0-__∞_ (ng*h/mL)	106.7	206.1
Vz (L/kg)	0.73	0.59
Vss (L/kg)	0.38	0.5
CL (L/(kg*h))	0.47	0.24

**Table 4:** Percentage LVEF measurements for Groups 1, 2, 3, 4, and 5 at various timepoints.

Left Ventricular Ejection Fraction (LVEF) Shown as %					
	Group 1 (Control)	Group 2 (AT1-HSA-MRN-NPs—i.v. Injection)	Group 3 (MRN Lactate—i.v. Injection)	Group 4 (Empty NPs)	Group 5 AT1-HSA-MRN-NPs—Subcutaneous Injection)
Baseline—Pre-Ligation	88.8 ± 2.1	89.9 ± 1.3	88.2 ± 1.8	88.4 ± 0.9	74.9 ± 1.7
48 h Post Ligation	69.1 ± 2.0	66.1 ± 1.9	65.3 ± 2.3	69.1 ± 2.2	66.4 ± 3.3
60 min Post Injection	68.6 ± 2.2	84.3 ± 2.9 ***	80.1 ± 3.3 **	70.1 ± 2.3	79.2 ± 2.7 **
24 h Post Injection	67.1 ± 2.1	84.2 ± 4.8 **	70.6 ± 3.6	67.5 ± 2.5	75.5 ± 2.6 **
48 h Post Injection	67.7 ± 1.8	83.1 ± 3.6 **	64.8 ± 4.3	64.9 ± 2.7	72.7 ± 2.3
1-week Post Injection	66.7 ± 1.1	74.5 ± 2.1 **	66.1 ± 4.2	67.2 ± 2.9	73.3 ± 2.9 *

The data have been presented as mean ± SD (*n* = 5). *** *p* < 0.001, ** *p* < 0.01, * *p* < 0.05 were considered significant based on Tukey’s post hoc analysis. The comparisons are with respect to the control Group 1.

**Table 5:** Percentage LVFS measurements for Groups 1, 2, 3, 4, and 5 at various timepoints.

Left Ventricular Fractional Shortening (LVFS) Shown as %					
	Group 1 (Control)	Group 2 (AT1-HSA-MRN-NPs—i.v. Injection)	Group 3 (MRN Lactate—i.v. Injection)	Group 4 (Empty NPs)	Group 5 AT1-HSA-MRN-NPs—Subcutaneous Injection)
Baseline—Pre-Ligation	52.6 ± 2.9	53.6 ± 2.1	52.6 ± 2.7	51.1 ± 1.3	54.6 ± 2.2
48 h Post Ligation	33.1 ± 1.5	29.8 ± 1.0	34.3 ± 2.7	31.2 ± 1.3	30.7 ± 2.5
60 min Post Injection	32.8 ± 1.7	50.1 ± 3.8 **	45.6 ± 3.2 **	34.7 ± 4.0	47.1 ± 3.9 **
24 h Post Injection	31.5 ± 1.2	50.4 ± 3.1 ***	33.6 ± 3.9	30.9 ± 2.4	40.5 ± 2.8 **
48 h Post Injection	31.7 ± 1.0	46.5 ± 3.1 **	30.2 ± 3.9	30.1 ± 1.5	34.8 ± 1.7
1-week Post Injection	31.3 ± 1.5	37.0 ± 2.7	30.8 ± 2.8	29.6 ± 1.1	35.9 ± 3.1

The data have been presented as mean ± SD (*n* = 5). The data have been presented as mean ± SD (*n* = 5). *** *p* < 0.001, ** *p* < 0.01, were considered significant based on Tukey’s post hoc analysis. The comparisons are with respect to the control Group 1.

## Data Availability

The data presented in this study to support the findings are available within the study.
